# 

**Published:** 1895-06-08

**Authors:** 


No. 454. Vol XVIII.

Saturday, June 8th, 1895.

WITH EXTRA NURSING SUPPLEMENT.

[Registered at the General Post Office as a Newspaper for transmission in the United Kingdom and Abroad.]

PRICE TWOPENCE.

Per Annum, 10s. 6d., post free.

Monthly Parts, 6d.; post free, 8d.

Ditto per Annum, 8s. 6d., post free.

